# Supplementary material for: Deletion of wfs1 Impairs Oligodendrocyte Precursor Cells Dorsal Distribution and Myelination Through the wfs1-hmgcs1 Axis in Zebrafish
Source: Biology (Basel). 2026 Aug 16;15(16):1402. doi: 10.3390/biology15161402 (PMC13510151; doi:10.3390/biology15161402)
Supplement: Supplementary file 1 [file biology-15-01402-s001.zip › File S1-Transcriptome sequencing data and differentially expressed genes (DEGs) in wfs1-- zebrafish at 5 dpf/2.KEGG/wfs1KOvsWT/GSEA/wfs1KO/PYRUVATE_METABOLISM/PYRUVATE_METABOLISM(DRE00620).html]

Details for gene set PYRUVATE\_METABOLISM(DRE00620)[GSEA]

|  || Dataset | deg.sample |
| Phenotype | sample.cls |
| Upregulated in class | wfs1KO |
| GeneSet | PYRUVATE\_METABOLISM(DRE00620) |
| Enrichment Score (ES) | 0.40108305 |
| Normalized Enrichment Score (NES) | 1.4974054 |
| Nominal p-value | 0.030864198 |
| FDR q-value | 0.19966613 |
| FWER p-Value | 0.652 |
Table: GSEA Results Summary

  

Fig 1: Enrichment plot: PYRUVATE\_METABOLISM(DRE00620)      
 Profile of the Running ES Score & Positions of GeneSet Members on the Rank Ordered List

  

| PROBE | DESCRIPTION (from dataset) | GENE SYMBOL | GENE\_TITLE | RANK IN GENE LIST | RANK METRIC SCORE | RUNNING ES | CORE ENRICHMENT || 1 | 116517 | adh5 |  |  | 148 | 4.502 | 0.0713 | Yes |
| 2 | 445233 | me2 |  |  | 202 | 4.186 | 0.1402 | Yes |
| 3 | 335715 | mdh1ab |  |  | 215 | 4.103 | 0.2091 | Yes |
| 4 | 406473 | pck2 |  |  | 487 | 3.031 | 0.2517 | Yes |
| 5 | 406702 | pdha1a |  |  | 691 | 2.614 | 0.2894 | Yes |
| 6 | 368239 | aldh2.2 |  |  | 1221 | 1.881 | 0.3045 | Yes |
| 7 | 445326\_1 | akr1a1a |  |  | 1428 | 1.705 | 0.3268 | Yes |
| 8 | 447920 | aldh3a2b |  |  | 1830 | 1.444 | 0.3385 | Yes |
| 9 | 58068 | pcxb |  |  | 1914 | 1.406 | 0.3596 | Yes |
| 10 | 559403\_1 | acaca |  |  | 1982 | 1.374 | 0.3807 | Yes |
| 11 | 114551 | pklr |  |  | 2861 | 1.068 | 0.3711 | Yes |
| 12 | 103909985 | LOC103909985 |  |  | 3098 | 1.013 | 0.3808 | Yes |
| 13 | 436914 | acss2 |  |  | 3398 | 0.954 | 0.3875 | Yes |
| 14 | 30643 | acat2 |  |  | 4174 | 0.798 | 0.3766 | Yes |
| 15 | 436672 | pdha1b |  |  | 4206 | 0.792 | 0.3889 | Yes |
| 16 | 100536873 | acot12 |  |  | 4243 | 0.787 | 0.4011 | Yes |
| 17 | 559403 | acaca |  |  | 5108 | 0.666 | 0.3852 | No |
| 18 | 100005587 | aldh9a1a.1 |  |  | 5134 | 0.663 | 0.3956 | No |
| 19 | 571263 | acyp1 |  |  | 6391 | 0.548 | 0.3653 | No |
| 20 | 399481 | aldh9a1b |  |  | 6718 | 0.518 | 0.3638 | No |
| 21 | 436747 | ldhbb |  |  | 6973 | 0.494 | 0.3642 | No |
| 22 | 336977 | hagh |  |  | 7212 | 0.473 | 0.3647 | No |
| 23 | 402939 | zgc:77938 |  |  | 7452 | 0.457 | 0.3649 | No |
| 24 | 436672\_1 | pdha1b |  |  | 8125 | 0.417 | 0.3508 | No |
| 25 | 393462 | aldh2.1 |  |  | 8615 | 0.394 | 0.3421 | No |
| 26 | 100000026 | aldh3a1 |  |  | 8825 | 0.379 | 0.3419 | No |
| 27 | 322482 | adh8a |  |  | 8920 | 0.373 | 0.3452 | No |
| 28 | 402985\_1 | grhprb |  |  | 9363 | 0.343 | 0.3371 | No |
| 29 | 335817 | pkma |  |  | 9917 | 0.309 | 0.3250 | No |
| 30 | 768196 | me1 |  |  | 10781 | 0.256 | 0.3022 | No |
| 31 | 393938 | fh |  |  | 10925 | 0.248 | 0.3019 | No |
| 32 | 402841\_1 | adh8b |  |  | 11968 | 0.193 | 0.2724 | No |
| 33 | 541435\_1 | acss1 |  |  | 12397 | 0.169 | 0.2618 | No |
| 34 | 30497 | ldhba |  |  | 13522 | 0.114 | 0.2284 | No |
| 35 | 492710 | aldh16a1 |  |  | 14167 | 0.084 | 0.2096 | No |
| 36 | 799805\_1 | akr1a1b |  |  | 15001 | 0.046 | 0.1842 | No |
| 37 | 334208 | ldhd |  |  | 15325 | 0.031 | 0.1745 | No |
| 38 | 402841 | adh8b |  |  | 16797 | -0.026 | 0.1288 | No |
| 39 | 101886626 | acyp2 |  |  | 17055 | -0.035 | 0.1213 | No |
| 40 | 402985 | grhprb |  |  | 17657 | -0.061 | 0.1034 | No |
| 41 | 556236 | acacb |  |  | 18028 | -0.077 | 0.0931 | No |
| 42 | 553283 | pcxa |  |  | 18484 | -0.098 | 0.0804 | No |
| 43 | 378727 | pck1 |  |  | 19075 | -0.125 | 0.0640 | No |
| 44 | 399662 | mdh1aa |  |  | 19301 | -0.135 | 0.0592 | No |
| 45 | 324201 | dlat |  |  | 19665 | -0.151 | 0.0504 | No |
| 46 | 406405 | mdh2 |  |  | 20457 | -0.185 | 0.0286 | No |
| 47 | 406428 | pdhb |  |  | 20690 | -0.198 | 0.0247 | No |
| 48 | 334197 | aldh7a1 |  |  | 21094 | -0.221 | 0.0157 | No |
| 49 | 445290 | acat1 |  |  | 21467 | -0.239 | 0.0081 | No |
| 50 | 30496 | ldha |  |  | 22490 | -0.295 | -0.0191 | No |
| 51 | 541435 | acss1 |  |  | 22524 | -0.297 | -0.0151 | No |
| 52 | 368213 | glo1 |  |  | 22941 | -0.323 | -0.0227 | No |
| 53 | 492719 | me3 |  |  | 23069 | -0.332 | -0.0211 | No |
| 54 | 100006238 | aldh9a1a.2 |  |  | 23161 | -0.338 | -0.0182 | No |
| 55 | 399479 | dldh |  |  | 25027 | -0.448 | -0.0693 | No |
| 56 | 799805 | akr1a1b |  |  | 25680 | -0.501 | -0.0813 | No |
| 57 | 560020 | acss2l |  |  | 26041 | -0.533 | -0.0837 | No |
| 58 | 323653 | aldh3a2a |  |  | 28024 | -0.745 | -0.1334 | No |
| 59 | 116517\_1 | adh5 |  |  | 28219 | -0.775 | -0.1264 | No |
| 60 | 497183 | grhpra |  |  | 29048 | -0.931 | -0.1367 | No |
| 61 | 406473\_1 | pck2 |  |  | 29767 | -1.153 | -0.1398 | No |
| 62 | 445094 | pkmb |  |  | 29848 | -1.183 | -0.1224 | No |
| 63 | 100006238\_1 | aldh9a1a.2 |  |  | 29971 | -1.231 | -0.1055 | No |
| 64 | 445326 | akr1a1a |  |  | 30087 | -1.279 | -0.0875 | No |
| 65 | 445233\_1 | me2 |  |  | 30440 | -1.469 | -0.0738 | No |
| 66 | 393427 | zgc:63568 |  |  | 31073 | -2.050 | -0.0591 | No |
| 67 | 100005587\_1 | aldh9a1a.1 |  |  | 31308 | -2.447 | -0.0252 | No |
| 68 | 100536795 | wu:fb14d09 |  |  | 31372 | -2.574 | 0.0163 | No |
Table: GSEA details [plain text format]

  

Fig 2: PYRUVATE\_METABOLISM(DRE00620)      
 Blue-Pink O' Gram in the Space of the Analyzed GeneSet

  

Fig 3: PYRUVATE\_METABOLISM(DRE00620): Random ES distribution      
 Gene set null distribution of ES for **PYRUVATE\_METABOLISM(DRE00620)**
